# The Impact of Smartphone Use on Brain Function in Adolescence: A Scoping Review

**DOI:** 10.3390/pediatric18020043

**Published:** 2026-03-17

**Authors:** Abby Marks, Meghan Berthelot, Hana Jones, Anna Kate Taylor, Karis Chang, Sydney Crozier, Sharon M. Cosper

**Affiliations:** Department of Occupational Therapy, Augusta University, Augusta, GA 30906, USA

**Keywords:** adolescence, smartphones, brain functioning, psychological disturbances, sleep, socioemotional functioning, executive functioning, sensory processing

## Abstract

*Background/Objectives*: The proportion of teenagers with access to a smartphone has reached 89 percent, marking a large increase in access to technology. Adolescence is a period of neuroplasticity where functional, structural, and systemic changes occur. Teenagers have experienced more persistent feelings of sadness and suicidality in recent years than ever before. Given the changes in this generation of adolescents and because adolescence is a period of neuroplasticity, this study seeks to understand the effects of smartphone use in adolescence. *Methods*: This scoping review was guided by the Preferred Reporting Items for Systematic Reviews and Meta-Analyses extension for Scoping Reviews (PRISMA-ScR). A total of 104 articles met the criteria for inclusion. *Results*: Analysis of results revealed five key themes: Psychological Disturbances (*n* = 52), Sleep (*n* = 43), Socioemotional Function (*n* = 23), Executive Function (*n* = 14), and Sensory Processing (*n* = 1). *Conclusions*: Results suggest that smartphones have a variety of effects on adolescent brain function that are primarily negative. The results of this study can inform the general population about the ways in which smartphone usage affects adolescent brain functioning. Further research is warranted to determine a causal relationship between smartphone use and adolescent brain functioning.

## 1. Introduction

Since 2012, the proportion of teenagers with access to a smartphone has doubled to reach 89 percent, marking a drastic increase in accessibility to technology for the new generation of adolescents [1]. Having a smartphone has become vital because it provides fast access to information, efficient communication, and social engagement [2]. In fact, 69 percent of adolescents say that spending time on their phone makes it easier for them to pursue interests and hobbies, and 45 percent say smartphones allow them to perform better in school [3]. However, 38 percent of adolescents admit to spending too much time on their phones [3]. Research suggests a maximum of two hours per day should be spent behind a screen; however, the average time teens spend using a smartphone is more than three hours and 49 min per day [4,5]. Additionally, following the COVID-19 Pandemic, the percentage of adolescents who spent more than two hours a day behind a screen increased from nine to 69 percent [6].

Smartphones provide opportunities for many different types of activities, including shopping, entertainment, and connecting with others. However, the design of smartphones has been made to be compelling and difficult to put down once in use [7]. Some adolescents go on to develop nomophobia, which describes the fear felt when an individual does not have their phone or access to a phone [8]. While having a smartphone can allow connection to social support networks, it often leads to habits that can be difficult to combat once in adulthood [6].

The period of brain development during adolescence, which is defined by developmental psychologist Dr. Jeffrey Arnett as ages 10 to 18, has proven to be a crucial time for maturation and growth [9,10]. It is known to be a period of significant change and neuroplasticity where both functional, structural, and systemic changes are occurring, specifically in the prefrontal cortex (PFC) and amygdala [11]. This period in human brain development is proven to affect humans’ decision making, impulse control, reasoning, stress levels, and other vital functions [9,11].

The prefrontal cortex develops late in adolescence and undergoes synaptic and neuronal pruning as well as dendritic and axonal growth, which leads to changes in cognitive processing, reasoning, memory, and social-emotional functioning [12]. The amygdala’s role in the brain is to aid in emotional processing and social interactions [13]. The maturation of the amygdala allows for notably more emotional control and regulation due to decreased connectivity with areas of the prefrontal cortex [13]. This neural connectivity is prominent between ages nine and 17 and decreases during the young adult and adult years starting around age 21 to allow for a decrease in amygdala reactivity and arousal [13]. Another area that is still maturing during adolescence is the reward system located in the forebrain, which includes the nucleus accumbens and the ventral striatum [14]. These brain regions that control reward processing are more active during social engagement with peers [14]. This heightened reward sensitivity and amygdaloid arousal, coupled with lower inhibitory control in the PFC, lead to a vulnerability to engaging in risky behaviors. Furthermore, current research has demonstrated that thicker cortical regions are correlated with higher intelligence [15]. The key areas that predict high school academic performance and achievement include the left fusiform gyrus, which plays a crucial role in language learning and memory, and the bilateral insula, which is important for selective attention, awareness, and cognitive functions [15].

Adolescence is a time of heightened vulnerability across a myriad of developmental systems coinciding with the peak occurrence of psychopathology [16]. In recent years, teenagers have experienced more persistent feelings of sadness, hopelessness, suicidal ideation, and attempted suicides than ever before [17]. Additionally, this generation of teenagers has experienced increased school loneliness twice as much as past generations [18]. Further, adolescents spend less time socializing and participating in paid work after school and less time engaging in extracurricular and leisure activities than generations previous [19]. This includes sporting events, entertainment, and spending time with others. They are also driving, dating, and going out without their parents less than generations previous [18]. Finally, despite increased enrollment rates, there has also been an increase in drop-out rates amongst high school seniors since 2020, where they had previously been on the decline [20]. Academic performance in this age group has declined in both reading and mathematics for both public and private students, marking the largest decline in three decades [21]. However, it is not understood if smartphone use is related to the increased prevalence of these issues in adolescent development.

Given the many changes that have occurred in this generation of adolescents and the context of the adolescent period being a neuroplastic phase of development, this study seeks to further understand the effects of smartphone usage during this time frame and its implications on development [22]. The purpose of this study is to understand the impact of smartphone use on adolescent brain functioning.

## 2. Materials and Methods

### 2.1. Design

This scoping review is guided by the Preferred Reporting Items for Systematic Reviews and Meta-Analyses extension for Scoping Reviews (PRISMA-ScR) (See Supplementary Materials) [23]. Institutional Review Board approval was not required due to the nature of a scoping review. Additionally, the study protocol was not registered. The search was conducted in February 2025.

### 2.2. Search Strategy

The search strategy was executed in collaboration with a research librarian to CINAHL, PsychInfo, and PubMed databases. Limitations included filters for articles published in English and articles published between 2022 and 2025. Duplicate articles were removed from the search results across databases. A sample search strategy, which was entered into PubMed, is available in Table 1.

### 2.3. Screening

All six researchers collaborated to develop the following inclusion criteria: (1) at least 75 percent of participants are ages 10–18, (2) published in English, (3) published from the year 2022 or later, and (4) articles discuss smartphone use, not exclusively computer or technology use alone. Studies were excluded based upon the following criteria: (1) articles that are systematic or scoping reviews, (2) articles that are not peer-reviewed, and (3) articles that mention computers, technology use, or screen time, but not smartphone use specifically. Level of evidence was not considered due to the nature of available evidence on smartphone use in adolescence being primarily survey-based.

Title and abstract screening were conducted by three groups of two researchers. The first 10 articles were screened by all six researchers to establish calibration amongst the researchers as evidenced by at least 80 percent agreement. Each article was screened separately by two researchers to determine if the article should move forward to the full text review. In the event that consensus was not established for an article, the researchers met to discuss article eligibility. If a consensus could not be reached, all six researchers met to resolve the discrepancy as a group. Articles from the title and abstract review were organized using a Google Sheets spreadsheet. Those selected from the initial title and abstract review were subject to full-text screening using a similar procedure as the initial screening.

### 2.4. Data Extraction and Analysis

The previously established pairs of researchers worked together to extract the following data from the included studies: authors, year, study design, country, participant age, participant gender, main brain function measured, smartphone use measured, and main findings. The first three articles were done by all researchers to establish calibration in the data charting process. Remaining articles were evenly divided, and each researcher conducted an initial data extraction and charting for their assigned articles. After this was complete, researchers exchanged articles with their previously established pair so that a second tiered review could verify their findings and identify potentially omitted information. Findings were then organized and entered into a Google Sheets spreadsheet. Researchers collaborated on conducting a thematic analysis to identify major themes across included articles.

## 3. Results

**Figure 1:**
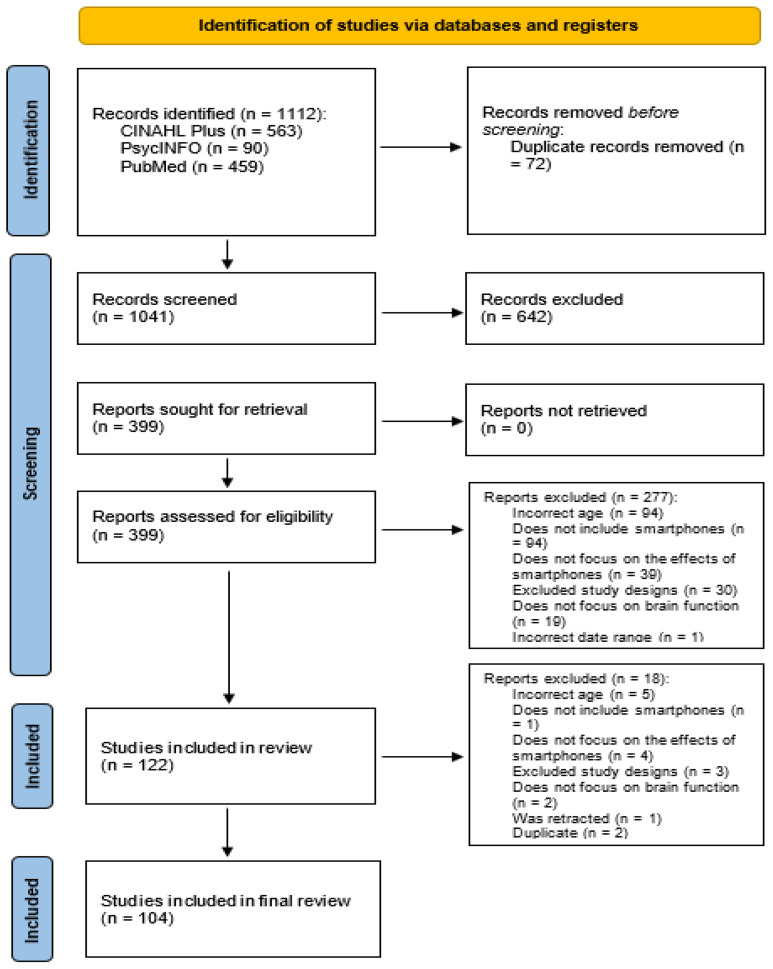
PRISMA flow diagram.

Figure 1 outlines the selection of studies using PRISMA-ScR. The 2025 search yielded 1112 results. After deduplication, the total number of articles remaining was 1041. After title and abstract screening, 399 articles remained. Following the full-text review, 122 articles remained and were included in the data charting process. An additional 18 articles were excluded during the data charting process. Reasons for exclusion throughout the review process included incorrect age range (*n* = 99), the study did not include smartphones in their study (*n* = 95), the study did not focus on the effects of smartphone use (*n* = 43), the study was not peer reviewed or was a review of the existing literature (systematic, scoping, or literature reviews) (*n* = 33), the study did not focus on a brain function as an outcome (*n* = 21), one study was excluded due to date of publication being out of established parameters for this study, one study was excluded due to it being retracted, and two studies were excluded due to being a duplicate. The total number of studies included in this scoping review was 104.

### 3.1. Characteristics of the Included Studies

**Figure 2:**
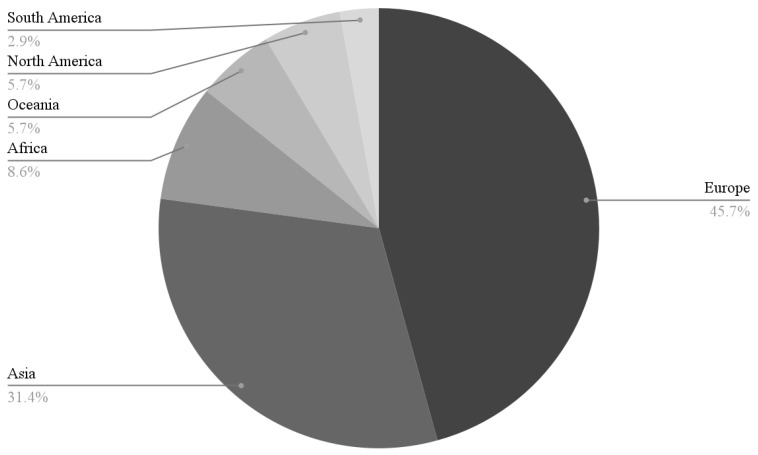
Distribution of countries represented by continent.

Included articles represent data collected from 37 different countries. The majority of included studies were conducted in China (*n* = 17), with other prevalent countries being the United States (*n* = 16), South Korea (*n* = 10), and Canada (*n* = 8). All continents, with the exception of Antarctica, are represented in this study (see Figure 2). The mean age of all reported means in the reviewed articles was 13.91 years. Male and female adolescents were represented fairly equally in the majority of included studies, with the exception of some studies being primarily male (*n* = 1) or female-focused (*n* = 8). The majority of study designs captured in this review included cross-sectional (*n* = 59), longitudinal survey designs (*n* = 16), and randomized controlled trials (RCTs) (*n* = 7). Other study designs included cohort studies, qualitative designs, and various mixed methods designs. The results of the studies provided information about the impact of smartphone use on the varying areas of brain functions.

### 3.2. Identified Themes

**Figure 3:**
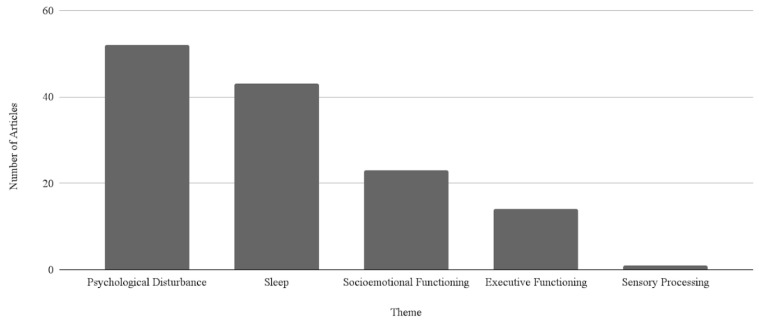
Established themes ordered by prevalence.

The purpose of this study was to understand the impact of smartphone use on adolescent brain functioning. Following data charting of the 104 included articles, a thematic analysis revealed five key themes and corresponding subthemes in order of prevalence: (1) psychological disturbance, (2) sleep, (3) socioemotional functioning, (4) executive functioning, and (5) sensory processing. See Figure 3 to view the identified themes ordered by prevalence. See Table 2 for the established subthemes, ordered by prevalence, within each identified theme.

### 3.3. Theme 1: Smartphone Impact on Psychological Disturbance

Fifty-two included articles discussed the correlation of smartphone usage, and especially problematic smartphone usage (PSU), with various forms of psychological disturbances. One study described PSU as a condition characterized by using a smartphone in an addictive, antisocial manner, leading to functional maladjustment [24]. Within this theme of psychological disturbance, five prevalent subthemes emerged, including: (1) depression, sadness, and suicidality, (2) anxiety and stress, (3) mental well-being and quality of life, (4) loneliness, and (5) body image distortion, disordered eating, and self-esteem.

#### 3.3.1. Subtheme 1: Depression, Sadness, and Suicidality

Of the 52 articles supporting the theme of smartphone usage’s effect on psychological disturbance, 30 discussed the relationship between smartphone usage and feelings of depression or sadness. These symptoms were found to be directly correlated to greater smartphone use [25,26,27]. However, certain types of smartphone usage, including using the camera, making calls, using streaming networks, using social networks, and sexting, resulted in greater instances of depressive symptoms [26,28,29]. In contrast, some articles found that using smartphones for messaging applications or for homework purposes was not related to depressive symptoms [29,30].

It is indicated that PSU in adolescents who rely heavily on their phones is linked to a stronger correlation with depressive symptoms [31,32,33,34]. For example, some research suggests that those who spend between 120 and 180 min using their smartphones display no depressive symptoms compared to their peers who spend more than 301 min using smartphones [35,36]. Some smartphone applications designed to target feelings of depression and sadness were found to be effective in reducing levels of depression, though findings suggest these reductions may be limited or temporary [37,38,39].

A total of five articles discussed suicidality as a psychological disturbance related to smartphone use. Adolescents with higher screen time, including on smartphones, displayed higher instances of suicidal ideation and suicide plans [34,40,41,42]. This relationship was stronger when the adolescent exhibited PSU or smartphone overdependence [34,42]. This pattern was also found to be stronger for males than for female adolescents [40,42].

#### 3.3.2. Subtheme 2: Anxiety and Stress

Of the articles supporting the theme of smartphones’ relationship to psychological disturbances in adolescents, 27 related to anxiety and/or stress. Experiences of both anxiety and stress are related to smartphone use, especially when smartphones are used to access social media sites [27,30,43,44,45,46]. However, those who exhibit addictive or problematic use behaviors related to their smartphone or to social media sites have a greater likelihood of experiencing stress or anxiety [25,33,47,48,49]. Five articles discussed the relationship between smartphone use and psychological distress, which involved feelings of anxiety and stress, depression, and self-harm behaviors [50]. Overall, higher smartphone usage was related to higher psychological distress, especially for adolescents who used their smartphones more than they desired to [24,50,51].

Contrastingly, some studies found that it was the absence of smartphones, including access to social networks, that induced anxious or stressed feelings [52,53]. Intentionally disconnecting from social media sites on smartphones increased stress for those who experience persistent urges to use their smartphone [53]. Further, one study suggested that social media sites including those accessed via smartphone were used to regulate feelings such as anxiety via access to social networks [53]. Similar to the relationship between smartphone use and depression, the amount of time spent using the smartphone is related to occurrence of stress and anxiety symptoms [35,36,49,54]. Greater than five hours spent using screens including smartphones was related to higher anxiety levels, whereas spending less than three hours per day using smartphones was correlated to more mild symptoms [49,55].

#### 3.3.3. Subtheme 3: Mental Well-Being and Quality of Life

Among the studies exploring smartphone-related psychological disturbances, 17 investigated impacts on mental well-being, overall mental health, and quality of life (QoL) in adolescents. Overdependence on smartphones is associated with poorer academic outcomes, decreased subjective health, and a general decline in emotional functioning [32,55]. These effects appear to be particularly strong in adolescents who use their phones excessively for non-relational purposes, such as escapism or habitual scrolling.

Gender differences also play a key role in how smartphone use affects well-being. Anthony et al. [56] found that frequent communication with virtual friends, rather than real-life peers, was associated with lower well-being, especially in girls. Researchers suggest that virtual friendship may lack the emotional depth that adolescent girls often seek, leading to unmet social needs and reduced emotional satisfaction. Similarly, girls who exceeded five hours of daily screen use reported significantly lower QoL scores compared to boys [54].

Although some adolescents report subjective benefits from using mental health apps, actual improvements in well-being are inconsistent. While Badesha et al. [57] noted an increase in emotional insight among participants, no significant changes in well-being or psychological flexibility were observed. Likewise, Zhou et al. [39] found that the Coping Camp app helped reduce stress and depressive symptoms but did not enhance overall mental well-being or coping skills. However, more structured interventions show promise. Raknes et al. [38] demonstrated that the HH app, which targeted emotional regulation and self-awareness, led to measurable improvements in adolescents’ well-being and emotional problem-solving.

#### 3.3.4. Subtheme 4: Loneliness

Among the studies examining smartphone-related psychological disturbances, five specifically addressed adolescents’ feelings of loneliness. Du et al. [58] found that adolescents experiencing loneliness often turned to their phones as a form of escape, resulting in a cycle of mobile phone addiction (MPA) and emotional suppression. This behavior stems from a perceived gap between the desired and actual levels of social connection. Rather than improving meaningful social engagement, increased phone use may reinforce isolation, further diminishing emotional well-being.

Patterns of digital multitasking during in-person interactions can also contribute to feelings of disconnection. One study identified adolescents who frequently engaged in digital social multitasking (DSMT) during face-to-face interactions, categorized as “embracers”, reported both high friendship quality and high levels of loneliness and digital stress [59]. In contrast, adolescents who were more intentional with their smartphone use experienced fewer negative symptoms and reported stronger social satisfaction.

Smartphone overdependence also appears to be a consistent factor in adolescent loneliness. Kim et al. [60] found that those classified as overdependent users reported significantly higher levels of loneliness compared to their non-dependent peers. Adolescents struggling with body image and high screen time, especially females, were also more likely to experience loneliness alongside other psychological stressors [45]. Although a direct link between PSU and loneliness was not found in all contexts, screen use exceeding five hours daily was related to increased loneliness and lower well-being overall [42].

#### 3.3.5. Subtheme 5: Body Image Distortion, Disordered Eating, and Self-Esteem

Among the studies examining smartphone-related psychological disturbances, five specifically addressed adolescents’ body image distortion, disordered eating, and self-esteem. Excessive smartphone and social media use among adolescents is strongly associated with body image dissatisfaction, disordered eating behaviors, and reduced self-esteem [35,45,61,62]. Multiple studies have identified that prolonged smartphone use, particularly for social networking or appearance-based gaming, correlates with distorted body image and unhealthy weight control practices [35,61]. It was found that greater time spent on social media and gaming predicted lower self-esteem and increased eating-related symptoms, underscoring a clear link between digital engagement and internalized appearance pressures [62]. These effects are more pronounced in adolescent girls, who tend to use their phones for longer durations and are more likely to engage with content that reinforces body comparison [45]. Smartphone overuse was also tied to inappropriate weight loss attempts, such as excessive physical activity or restrictive dieting, especially among those using apps focused on appearance or messaging [61]. Furthermore, Engberg et al. [44] reported that higher digital media use was negatively associated with self-esteem and physical activity.

### 3.4. Theme 2: Smartphone Impact on Sleep

Forty-three included articles discussed the correlation of smartphone usage with sleep. Within the theme of sleep, four subthemes emerged, including: (1) sleep quality, (2) sleep duration and disturbances, (3) sleep onset, (4) daytime functioning, and (5) social jetlag.

#### 3.4.1. Subtheme 1: Sleep Quality

Of the 43 articles supporting the theme of smartphone usage’s effect on sleep, 25 discussed the relationship between smartphone usage and sleep quality. Research indicates that an overdependence on one’s smartphone is directly correlated with insufficient sleep [31,63]. Smartphone overdependence is related to poor impulse control, making it difficult to put down the smartphone once in use, thus causing poor sleep quality amongst this population [63].

Multiple studies found that adolescent girls are more likely to suffer from poor sleep quality than boys, as well as those who spend more than eight hours per day using their smartphones [64,65,66]. Additionally, social media use before bed was commonly found to have negative outcomes on sleep quality [67,68,69]. Adolescents who spent time communicating with peers and engaging in social activity (e.g., texting, calling, commenting on social media posts, etc.) were found to have poorer sleep outcomes than engaging in fewer social activities [67,69,70].

#### 3.4.2. Subtheme 2: Sleep Duration and Sleep Disturbances

Of the 43 articles supporting the theme of adolescent smartphone use on sleep, 22 specifically discussed the relationship of smartphone use with sleep duration and sleep disturbances. The majority of findings suggest that the consequences of smartphone use in differing forms, including social media, texting, and calling leads to overall shorter sleep duration [67,71,72]. One specific way adolescents are using their smartphones is to view social media [68,73,74]. Although most findings report the negative consequences of smartphone use on sleep duration, one article found positive consequences within its findings [75]. This article finds that adolescent smartphone use before bed led to longer sleep duration patterns in adolescents observed [75]. Similar to the findings on sleep duration, the evidence supporting the relationship between adolescent smartphone use and sleep disturbances showed comparable results. Some of these findings suggest that adolescents using their smartphones immediately before bedtime were linked to sleep disturbances [65,73]. Specifically, multiple studies found that adolescents who used their smartphones at night in bed experienced difficulties falling and staying asleep [65,73].

#### 3.4.3. Subtheme 3: Sleep Onset

Of the 43 articles supporting the theme of adolescent smartphone use on sleep, 17 specifically discussed the relationship between smartphone use and sleep onset. Smartphone use, especially immediately before going to bed, is highly indicative of later bedtimes. More specifically, those who spend time on their smartphone at least one hour before bedtime are at a higher risk of requiring more than 60 min to fall asleep each night [66]. Further research indicates that actively using one’s smartphone (e.g., communicating with others, playing video games, etc.) is a positive predictor of a later sleep onset as compared to passive engagement (e.g., watching a video, playing music, etc.) [69,74,76]. According to Daniels et al. [77], adolescents who use their phones at night as a distraction from negative thoughts and feelings often have a sleep onset time of up to 52 min later than those who do not. Consequently, blue light emission from smartphones results in a suppression of melatonin production, causing a delay in circadian rhythm amongst smartphone users [78].

Contrastingly, some research has found that adolescents enjoy using their smartphones for relaxation purposes to assist in falling asleep, such as listening to music or watching relaxing videos. In fact, those adolescents feel as though they are dependent on their smartphone as it becomes harder to fall asleep without it [79]. Additional findings note that using one’s smartphone for academic purposes is not directly correlated to later sleep onset [80]. Ultimately, there are a variety of ways in which one can utilize their smartphone, creating both positive and negative effects on sleep onset.

#### 3.4.4. Subtheme 4: Daytime Functioning

Of the 43 articles supporting the theme of adolescent smartphone use on sleep, four specifically discussed the relationship between smartphone use and daytime functioning. PSU, especially before bed, is directly correlated with higher levels of daytime sleepiness, thus making it difficult for adolescents to experience optimal functioning during the day [70,79]. Specifically, Li et al. [81] report that PSU is a key predictor of disengagement during school hours and academic procrastination. Consequently, there is a direct correlation between PSU, poor sleep quality, and school disengagement. As stated previously, longer duration of screen time is related to poor sleep quality [82,83]. In turn, a lack of sufficient sleep makes it difficult for adolescents to maintain a balanced mental state throughout the day [81]. Some adolescents even report experiencing difficulty managing their emotions and interpersonal relationships when they have had inadequate sleep [79].

Research also suggests that PSU is an indicator of challenges with daytime functioning, especially for girls or for those who use their smartphones at least three nights per week [70]. Additionally, social media use is one of the most common ways in which one engages with their smartphone and is found to be closely associated with excessive daytime sleepiness [67]. Further, Chaveepojnkamjorn et al. [67] found that using one’s smartphone to engage in social networks, send texts or emails, and call peers for more than two hours a day can increase experiences of daytime sleepiness and impair optimal functioning throughout the day.

#### 3.4.5. Subtheme 5: Social Jetlag

Of the 43 articles supporting the theme of adolescent smartphone use on sleep, four specifically discussed the relationship of smartphone use with social jetlag. Social jetlag is a discrepancy in the human body’s internal perception of biological versus social time [84]. Studies investigating social jetlag compared the sleep patterns of adolescents on weekdays versus weekends [78,84]. Results found that adolescents experienced increased social jetlag when they had a screen time of at least six hours [85]. Specifically, it was found that adolescents who use their smartphones for at least four hours per day, with an increased usage time on the weekends, resulted in overall higher social jetlag than adolescents with an increased usage time on the weekdays [85]. The studies consistent with all these findings were conducted in Southeast Asian countries such as Japan, China, and South Korea [78,84,85].

### 3.5. Theme 3: Smartphone Impact on Socioemotional Functioning

Twenty-three included articles discussed the impacts of smartphones on adolescent socioemotional functioning, operationalized as their ability to engage in processes required for both social and emotional domains with others and themselves [86]. Within this broad theme, three main subthemes emerged, including: (1) emotional regulation, (2) social connectedness and emotional support, and (3) social participation.

#### 3.5.1. Subtheme 1: Emotional Regulation

From the 23 total articles assessing the correlations of smartphone use and socioemotional functioning, 13 of them described emotional regulation as a major theme. Emotional regulation typically refers to emotion-driven self-control, managing stress and anxiety, and anger reactivity [52,87,88]. Smartphones and social media are used by adolescents as a way to regulate their emotions, but research shows that using phones for this reason can be both beneficial and harmful [53].

Apps on smartphone devices have been used as emotional regulation tools by researchers and were shown to improve emotional regulation skills, including the ability to both identify and understand various emotions and thought patterns [38,89]. An intervention app was used on the smartphones in those aforementioned studies, leading to increased emotional regulation skills, but most other research finds that smartphone use for general and entertainment use led to difficulty managing emotions [24,88].

Individuals using their devices for socializing and playing competitive games for longer than three hours per day reported higher levels of anger in addition to having difficulty controlling it, and using their devices for longer than two hours led to higher levels of impulsivity regardless of sleep and emotional well-being [88,90]. Adolescents overdependent on their mobile devices suffer from increasingly diminished emotional regulation skills as they continue to overuse them [50]. Increased impulsivity and decreased self-control are seen as studies show they experience frequent urges to pick up their smartphones and oftentimes are unaware of the physical act; therefore, they find themselves unable to control how extensively they use their phones [50,53]. Adolescents engaging in PSU use their devices as a strategy for filling an emotional void and are directly associated with rumination, a key aspect of emotional dysregulation, leading to a cyclical pattern of negative thoughts without having the skills to manage or problem-solve them [24,38].

#### 3.5.2. Subtheme 2: Social Connectedness and Emotional Support

Ten articles described the impacts of smartphone use on the social connectedness of adolescents. Females were found to use smartphones for social and emotional reasons, such as checking social media, more frequently than males, who typically utilize them for entertainment purposes such as gaming [91,92]. For both genders alike, adolescents use smartphones and social media to stay informed about recent events, connect with others and social network, combat their fear of missing out (FOMO), and gain social acceptance with their peers [35,53]. Because a large portion of adolescents use their smartphones as a way to stay connected, adolescents report higher levels of social connectedness, peer support, and sense of community [91,93]. Not all social connection was positive, though. Adolescents expressed that they felt compelled to use their phones, overwhelmed by peer pressure and the need to conform to societal expectations, which lead to negative social comparison; conversely, not using their phones led to feelings of loneliness, disconnection, and anxiousness [52,94].

Some adolescents, specifically those with PSU or MPA, report feeling less social support and develop negative attachment styles with their caregivers [92]. They also feel lonelier and resort to using their phone as a way to escape, leading to a positive feedback loop where PSU leads to decreased feelings of social connection and phones are used as a form of escapism, creating an increasing gap between desired and actual levels of social connection [58]. One study also found that spending time away from smartphones and social media led females to report higher levels of social connection and more time spent engaging in activities with others [95].

One article specifically addressed its effects on emotional support. This article highlighted the impact of adolescents with MPA and its correlation to a lack of strong social support and experiencing negative attachment to caregivers, leading them to rely on their phones for emotional comfort and connection [92]. It was posited that this emotional dependence, particularly common among girls, leads adolescents to use their phones at night to cope with distress [92].

#### 3.5.3. Subtheme 3: Social Participation

Out of the 23 articles examining the prevalence of smartphone use and its impact on adolescents’ socioemotional functioning, two articles specifically explored how smartphone use affects adolescent social participation. Both studies focused on how social participation through digital mediums affect adolescents’ mental health and social participation [96,97]. Mao and colleagues [97] found that an app-based training to be used on smartphones helped reduce social anxiety and fear of negative evaluation, potentially enhancing adolescents’ ability to engage more confidently in social situations. Meanwhile, Brand and colleagues [96] showed that excessive or addictive use of social networking sites, especially when it disrupts daily social activities, can hinder healthy social participation.

### 3.6. Theme 4: Smartphone Impact on Executive Functioning

Within our search, 14 articles discussed the correlation of smartphone use on executive functioning in adolescents. Existing evidence suggested that there were intensive studies and current research occurring to examine the consequences of adolescents’ exposure to screens and how excessive screen exposure can be detrimental to adolescents’ cognitive health and overall well-being [98]. Within this theme of executive functioning, four subthemes emerged, including: (1) attention, (2) impulse control, (3) language and memory, (4) decision making, and (5) critical thinking. These subthemes correlate to different forms of executive functioning consequences that emerged within this overarching theme.

#### 3.6.1. Subtheme 1: Attention

Of the 14 articles examining the impact of smartphone use on executive functioning in adolescents, four specifically explored how smartphone use affects attention-related outcomes. Across these four studies, higher levels of screen time—especially involving social media, video games, and mobile phone use—were consistently linked to attention difficulties in adolescents [98,99,100,101]. A few studies found that while social media use did not impact overall cognitive abilities, it was linked to attention-specific challenges, with video games, texting, and educational technology associated with attention deficits [99,100]. Poujol and colleagues [98] also noted a link between mobile screen time and increased inattentiveness in otherwise healthy adolescents. Wallace et al. [101] further supported these findings, showing that greater use of social media, television, and video games is thought to be predictive of higher ADHD symptoms, largely driven by weakened attention regulation.

#### 3.6.2. Subtheme 2: Impulse Control

Among the 14 articles examining smartphone use and executive functioning in adolescents, four articles specifically addressed the impact of smartphone use on impulse control. Across these four studies, problematic smartphone and media use in adolescence was linked to poor impulse control and self-regulation [87,90,101,102]. Some articles found that high screen time, especially being on social media for more than two hours daily, was associated with increased impulsivity and decreased regulation [90,101]. Additionally, it was shown in other articles that low self-control may contribute to compulsive phone use and overlapped with behavioral regulation challenges, highlighting impulsivity as a key risk factor in excessive media use [87,102].

#### 3.6.3. Subtheme 3: Language and Memory

Of the total articles concerning executive functioning in adolescents, four articles discuss smartphones’ effects on specific cognitive functions, including verbal intelligence, focus, spatial perception, information processing, creativity, working memory, and attention [43,100]. Overall, using smartphones and electronic devices was found to be correlated with lower cognitive scores [43]. PSU was found to be linked to poorer cognitive performance, specifically for memory tasks such as learning and recalling word pairs [103]. Researchers identified that adolescents who regularly used their smartphones and media were less able to consolidate their memories into retrievable information. Adolescents who actively use their smartphones and engage with content have slightly lower verbal intelligence skills compared to those who use their phones passively, simply watching the screen without interacting with media [100]. While adolescents’ short-term working memory might be impacted by using social media and smartphones, these researchers did not find a lasting effect or any meaningful association on memory, intelligence, information processing, or creativity, only on practical numeracy [100].

#### 3.6.4. Subtheme 4: Decision Making

Three of the 14 total articles provided correlates about poor decision making habits that impact their daily functioning and various domains of health. Some of these decisions include engaging in risky behaviors, dangerous weight loss habits, sexting, and delinquent behaviors [35,104]. One article found that adolescents overdependent on their smartphones were linked to higher engagement in risky lifestyle behaviors, including poor diet, low physical activity, poor sleep habits, increased alcohol consumption, and smoking [34]. Another study highlights the relationship between adolescents engaging in delinquent behaviors, including substance use (e.g., alcohol, marijuana, and other drugs), property damage, violent acts, and sexual behaviors, with increased smartphone use, with the connection being particularly strong among adolescents who participate in sexting [104].

One article focused on the impact of adolescent smartphone use on critical thinking, which impacts decision making [105]. This study found that high smartphone users perform worse on critical thinking assessments compared to low users. They were found to exhibit significantly poorer scores for critical analysis, inference, clarification, evaluation, and self-monitoring on both the social and mathematical problems provided to them to complete. All of these domains assessed contribute to an adolescent’s ability to think critically and are shown to be negatively correlated with high smartphone usage.

### 3.7. Theme 5: Smartphone Impact on Sensory Processing

One included article discussed the correlation of smartphone usage with sensory processing. This study defined sensory processing as the method by which the nervous system organizes and interprets information received from the senses [106]. The findings suggest that PSU is directly correlated to difficulties with sensory processing and may contribute to sensory overload amongst typically developing adolescents. On the contrary, there are no correlations between smartphone use and sensory processing amongst adolescents with ADHD.

## 4. Discussion

In this scoping review, we identified five major areas of adolescent brain function related to smartphone use. The findings reveal a deeper understanding of the impact smartphone use can have on various brain functions in adolescence, including links to psychological disturbances, sleep, socioemotional function, executive function, and sensory processing. These identified themes relate to all major brain functions, and outcomes are primarily negative.

As previously stated, adolescence is a period of significant neuroplasticity, especially in the amygdala, prefrontal cortex, and occipital and temporal lobes [11,107]. This period in human brain development is crucial in developing decision making skills, impulse control, reasoning, stress levels, and other vital functions [9,11]. Based on the results of this scoping review, areas of the brain related to these brain functions may be impacted by smartphone use in adolescence.

The amygdala is known to aid in emotional control and regulation for emotions such as fear and anxiety [13]. Thus, the amygdala is compromised when an individual is experiencing depressive or anxious symptoms. Therefore, depression and anxiety linked to smartphone use may indicate abnormal functioning in the amygdala. It was found that adolescents who use their devices suffer from deficits in socioemotional functioning and have difficulty controlling emotions and often use their devices as a maladaptive emotional regulation strategy to escape their reality [58,88,90]. In addition to a form of escapism, adolescents used their smartphones as an emotional security blanket, being dependent on them and feeling compelled to use them [92]. Without their phones, they felt socially disconnected, anxious, and alone, revealing the possible impacts of smartphone use on the amygdala [52,94].

Further, the PFC is extensively pruned in adolescence, leading to changes in cognitive processing, reasoning, memory, and social-emotional functioning [12]. One of the identified themes, executive functioning, involves numerous cognitive processes, including reasoning, memory, and decision making, indicating potential negative changes in the PFC linked to smartphone use. Additionally, the reward system within the PFC is still maturing in adolescence and is found to be more active and sensitive, leading to lower inhibitory control [14]. This system can impact adolescents’ attention, impulse control, and decision making, all of which were found to be negatively associated with smartphone use [35,90,98,101,104]. This creates an amplification effect where the reward system that is already highly sensitive with a lower threshold required for decision making is coupled with increased smartphone use that lowers impulse control, leading to poor decision making [14,90,101]. The reward system is also especially active during social engagement with peers, so it can be deduced that engaging with peers online using smartphones activates the reward system and releases dopamine, further reinforcing the addictive effects of smartphones [14,108].

The left fusiform gyrus, housed in the occipital and temporal lobes, plays a role in learning and memory and is another brain structure that is undergoing significant developmental changes during the adolescent period [15,107]. PSU was associated with lower cognitive performance in adolescents, specifically in language and memory tasks, further showcasing the sensitivity of the fusiform gyrus and the magnification of detrimental effects that can happen when using smartphones improperly [103].

Some of the identified themes were found to be interrelated and influential on each other. For example, psychological disturbances such as depression and anxiety are related to poor sleep quality [36,74]. In addition, adolescents are found to use their smartphones as a means of emotional regulation; however, doing so results in staying up later to avoid negative thoughts and emotions [77]. Having a later bedtime and thus a shorter sleep duration has shown negative consequences during the day as adolescents report feeling difficulty concentrating and managing social connections [79,81]. Multiple studies found that smartphone overuse was related to various executive functioning processes including decreased impulse control that leads to poor decision making, but this limited self-control, resulting in the inability to cease smartphone use, was also found to be correlated with poorer sleep quality and insufficient sleep [63,87,102].

Studies show that excessive smartphone use in adolescents is closely tied to interconnected emotional, behavioral, and social challenges that often interact and reinforce each other. Spending several hours daily on devices for gaming or socializing is linked to increased anger, impulsivity, and difficulty managing emotions, all of which hinder social development [88,90]. Adolescents who overuse smartphones often struggle with self-control, feel compelled to check their devices, and, in turn, use them to fill emotional voids instead of seeking out physical interactions [50,53]. This poor impulse control related to smartphone dependence intensifies emotional instability, fosters rumination, and can be linked to disrupted peer interactions and decreased social participation [24,38,96]. Moreover, substituting face-to-face interaction with digital communication, especially through social media, disrupts social development and limits opportunities for meaningful connection, contributing to psychological disturbances such as loneliness and isolation [96]. These interlinked patterns show that excessive smartphone use impairs adolescents’ impulse control and emotional regulation, while simultaneously undermining their social functioning, creating a self-reinforcing cycle of psychological and behavioral difficulties.

The subthemes of mental well-being and quality of life, loneliness, and body image distortion appear to function in a mutually reinforcing cycle, compounding the psychosocial impact of PSU in adolescence. For instance, adolescents reporting poor body image and disordered eating behaviors often exhibit diminished self-esteem and greater loneliness, particularly among females who engage in appearance-focused digital content [45,61,62]. Loneliness, in turn, has been shown to predict increased reliance on smartphones as a form of emotional suppression and escapism, which may lead to heightened smartphone dependency and decreased emotional well-being [31,58]. Additionally, adolescents who primarily engage with virtual peers rather than in-person relationships report lower overall well-being, likely due to a lack of emotional depth and social fulfillment in digital interactions [55,56]. These interconnected psychosocial variables, low self-esteem, social isolation, and emotional avoidance, create a feedback loop that perpetuates psychological distress and diminishes perceived quality of life.

### 4.1. Limitations

One of the main limitations of this scoping review is the methodology of the scoping review process. The majority of studies included are cross-sectional or survey-based, so causal inference is strongly cautioned. The potential associations between smartphone use and the findings of this investigation being bidirectional is worth consideration. We also acknowledge the potential limitations of restricting our included articles to only English-language, peer-reviewed studies, as this may exclude valuable insights from the non-English and non-peer-reviewed literature and introduce the possibility of publication bias. Further restricting the date range to 2022–2025 potentially omitted relevant findings; this was done in an attempt to limit the influence of the Coronavirus Pandemic on smartphone use findings. Further, not all reviewers reviewed every article, which may lead to subjectivity and differing agreement, impacting the inclusion or exclusion of articles. Included articles were primarily of low levels of evidence, which leads to varying qualities of evidence, the inability to generalize findings, and an increased risk of bias. Additionally, studies were included even if they explored smartphone usage alongside other types of electronic devices such as computers, video gaming systems, and TVs. As a result, it may be difficult to isolate the specific impact of smartphone usage, given that the observed effects on adolescent brain development could be influenced by these other forms of technology.

### 4.2. Implications for Research

Further research is needed to determine causal relationships between smartphone use and adolescent brain functioning. The inclusion of neuroimaging approaches may be particularly helpful in examining this relationship and the extent of its possible impact on the adolescent brain. Although some of this literature does exist, more is necessary [109].

## 5. Conclusions

The purpose of this scoping review was to understand the impact of smartphone use on adolescent brain functioning. An extensive review of 104 included articles revealed five key themes related to brain functions in adolescence, including psychological disturbances, sleep, socioemotional function, executive function, and sensory processing. The results of this study align with previous findings on the topic and have various implications for clinicians, adolescents, and caregivers [110]. Clinicians should be aware of the deficits linked to smartphone use discussed in this study and implement interventions to promote optimal brain functioning outcomes. They should use this knowledge to educate their clients on the impacts of smartphone use on brain functioning, and especially PSU, based on the links to negative outcomes discussed previously. Adolescents should understand the negative impact of smartphone use on brain functioning in order to make informed decisions and form optimal habits relating to the use of their devices. Finally, caregivers should be aware of the potential consequences of smartphone use on their adolescents’ brain functioning and facilitate recommendations for screen time limitations, which is approximately two to three hours per day [49,54,88,90,101]. Thus, as adolescents’ access to smartphones rises to 89 percent of the adolescent population and will only continue to increase in the future, it is imperative that these prevalent physical and cognitive disturbances continue to be researched and disseminated to all individuals [1]. Ultimately, understanding the effects of smartphone usage on adolescent brain function highlights the vital role of clinicians in promoting balanced routines, facilitating emotional regulation, and supporting patients and caregivers through navigating the ever-growing digital world in a way that promotes healthy development and functional well-being.

## Figures and Tables

**Table 1:** PubMed Search Strategy.

Step	Search Query	Results
#1	“Adolescent” [Mesh] OR “Adolescent Development” [Mesh]	2,307,120
#2	“Smartphone” [Mesh] OR “Cell Phone” [Mesh] OR “Mobile Applications” [Mesh] OR “Social Networking” [Mesh] OR “Screen Time” [Mesh] OR “problematic smartphone use” OR “smartphone dependence” OR “smartphone use” OR “iPhone” OR “android” OR “mobile phone” OR “mobile phone addiction” OR “phone use”	55,019
#3	“Brain Mapping” [Mesh] OR “Brain” [Mesh] OR “Cerebrum” [Mesh] OR “Cerebral Cortex” [Mesh] OR “Frontal Lobe” [Mesh] OR “Prefrontal Cortex” [Mesh] OR “Parietal Lobe” [Mesh] OR “Amygdala” [Mesh] OR “Limbic Lobe” [Mesh] OR “Gyrus Cinguli” [Mesh] OR “Gray Matter” [Mesh] OR “Insular Cortex” [Mesh] OR “Neocortex” [Mesh] OR “Occipital Lobe” [Mesh] OR “Sensorimotor Cortex” [Mesh] OR “Temporal Lobe” [Mesh] OR “Executive Function” [Mesh] OR “Cognition” [Mesh] OR “Perception” [Mesh] OR “Problem Solving” [Mesh] OR “Attention” [Mesh] OR “Thinking” [Mesh] OR “Memory” [Mesh] OR “Sleep” [Mesh] OR “Motivation” [Mesh] OR “Emotional Regulation” [Mesh] OR “Social Skills” [Mesh] OR “Mental Health” [Mesh] OR terms related to prefrontal cortex and brain function	3,209,325
#4	“Internet Addiction Disorder” [Mesh]	1448
#5	#1 AND #2	8222
#6	#5 AND #3	1832
#7	#6 NOT #4	1803
--	Final Filter: English language, Publication Date 2022–2025	459

**Table 2:** Main themes and subthemes identified.

Main Themes	Subthemes
Psychological Disturbance	Depression, Sadness, and Suicidality
	Anxiety and Stress
	Mental Well-Being and Quality of Life
	Loneliness
	Body Image Distortion, Disordered Eating, and
	Self-Esteem
Sleep	Sleep Quality
	Sleep Duration and Sleep Disturbance
	Sleep Onset
	Daytime Functioning
	Social Jetlag
Socioemotional Functioning	Emotional Regulation
	Social Connectedness and Emotional Support
	Social Participation
Executive Functioning	Attention
	Impulse Control
	Language and Memory
	Decision Making
Sensory Processing	No Subthemes Identified

## Data Availability

No new data were created or analyzed in this study.

## Supplementary Materials

The following supporting information can be downloaded at: https://www.mdpi.com/article/10.3390/pediatric18020043/s1, File S1: Preferred Reporting Items for Systematic reviews and Meta-Analyses extension for Scoping Reviews (PRISMA-ScR) Checklist.

## References

[1] Abi-Jaoude E., Naylor K.T., Pignatiello A. (2020). Smartphones, social media use and youth mental health. Can. Med. Assoc. J..

[2] Barrick E.M., Barasch A., Tamir D.I. (2022). The unexpected social consequences of diverting attention to our phones. J. Exp. Soc. Psychol..

[3] Anderson M., Faverio M., Park E. (2024). How Teens and Parents Approach Screen Time.

[4] Sales de Lucena J.M., Cheng L.A., Cavalcante T.L.M., da Silva V.A., de Farias Júnior J.C. (2015). Prevalence of excessive screen time and associated factors in adolescents. Rev. Paul. Pediatr..

[5] Tomczyk Ł., Selmanagic Lizde E. (2023). Is real screen time a determinant of problematic smartphone and social network use among young people?. Telemat. Inform..

[6] Lua V.Y., Chua T.B., Chia M.Y. (2023). A narrative review of screen time and wellbeing among adolescents before and during the COVID-19 pandemic: Implications for the future. Sports.

[7] Radesky J.S., Kaciroti N., Weeks H.M., Schaller A., Miller A.L. (2022). Longitudinal associations between use of mobile devices for calming and emotional reactivity and executive functioning in children aged 3 to 5 years. JAMA Pediatr..

[8] Chang J. (2024). 90 Smartphone Addiction Statistics You Must See: 2024 Usage and Data Analysis.

[9] Aoki C., Romeo R.D., Smith S.S. (2017). Adolescence as a critical period for developmental plasticity. Brain Res..

[10] Arnett J.J. (2000). Emerging adulthood: A theory of development from the late teens through the twenties. Am. Psychol..

[11] Zimmermann K.S., Richardson R., Baker K.D. (2019). Maturational changes in prefrontal and amygdala circuits in adolescence: Implications for understanding fear inhibition during a vulnerable period of development. Brain Sci..

[12] Uytun M.C. (2018). Development period of prefrontal cortex. Prefrontal Cortex.

[13] Page C.E., Biagiotti S.W., Alderman P.J., Sorrells S.F. (2022). Immature excitatory neurons in the amygdala come of age during puberty. Dev. Cogn. Neurosci..

[14] Andrews J.L., Ahmed S.P., Blakemore S.J. (2021). Navigating the social environment in adolescence: The role of social brain development. Biol. Psychiatry.

[15] Meruelo A.D., Jacobus J., Idy E., Nguyen-Louie T., Brown G., Tapert S.F. (2018). Early adolescent brain markers of late adolescent academic functioning. Brain Imaging Behav..

[16] Sisk L.M., Gee D.G. (2022). Stress and adolescence: Vulnerability and opportunity during a sensitive window of development. Curr. Opin. Psychol..

[17] Centers for Disease Control (2024). Youth Risk Behavior Survey Data Summary & Trends Report: 2013–2023. https://www.cdc.gov/yrbs/dstr/index.html.

[18] Twenge J.M., Park H. (2017). The decline in adult activities among U.S. adolescents, 1976–2016. Child Dev..

[19] Livingston G. (2019). The Way U.S. Teens Spend Their Time Is Changing, but Differences Between Boys and Girls Persist.

[20] US Census Bureau (2020). Census Bureau Releases New School Enrollment Data. https://www.census.gov/newsroom/press-releases/2020/school-enrollment-data.html.

[21] National Assessment of Educational Progress NAEP Report Card: 2022. The Nation’s Report Card. https://www.nationsreportcard.gov/highlights/mathematics/2022/.

[22] Ismail F.Y., Fatemi A., Johnston M.V. (2017). Cerebral plasticity: Windows of opportunity in the developing brain. Eur. J. Paediatr. Neurol..

[23] Tricco A.C., Lillie E., Zarin W., O’Brien K.K., Colquhoun H., Levac D., Moher D., Peters M.D.J., Horsley T., Weeks L. (2018). PRISMA extension for scoping reviews (PRISMA-SCR): Checklist and explanation. Ann. Intern. Med..

[24] Arrivillaga C., Rey L., Extremera N. (2022). Psychological distress, rumination and problematic smartphone use among Spanish adolescents: An emotional intelligence-based conditional process analysis. J. Affect. Disord..

[25] Humer E., Probst T., Wagner-Skacel J., Pieh C. (2022). Association of Health Behaviors with mental health problems in more than 7000 adolescents during COVID-19. Int. J. Environ. Res. Public Health.

[26] Klinger D., Plener P.L., Marboe G., Karwautz A., Kothgassner O.D., Dienlin T. (2024). Exploring the relationship between media use and depressive symptoms among gender diverse youth: Findings of the Mental Health Days study. Child Adolesc. Psychiatry Ment. Health.

[27] Mohd Saat N.Z., Hanawi S.A., Hanafiah H., Ahmad M., Farah N.M., Abdul Rahman N.A. (2024). Relationship of screen time with anxiety, depression, and sleep quality among adolescents: A cross-sectional study. Front. Public Health.

[28] Frøyland L.R., Tokle R., Burdzovic Andreas J., Brunborg G.S. (2024). Sexting and mental health in adolescence: A longitudinal study. J. Adolesc. Health.

[29] Huang S., Lai X., Li Y., Cui Y., Wang Y. (2023). Beyond screen time: The different longitudinal relations between adolescents’ smartphone use content and their mental health. Children.

[30] Visier-Alfonso M.E., López-Gil J.F., Mesas A.E., Jiménez-López E., Cekrezi S., Martínez-Vizcaíno V. (2024). Does socioeconomic status moderate the association between screen time, mobile phone use, social networks, messaging applications, and mental health among adolescents?. Cyberpsychol. Behav. Soc. Netw..

[31] Kim N.-H., Lee J.-M., Yang S.-H., Lee J.-M. (2022). Association between smartphone overdependency and mental health in Korean adolescents during the Covid pandemic; age-and gender-matched study. Front. Public Health.

[32] Li Y., Wang Z., You W., Liu X. (2022). Core self-evaluation, mental health and mobile phone dependence in Chinese high school students: Why should we care. Ital. J. Pediatr..

[33] Mayerhofer D., Haider K., Amon M., Gächter A., O’Rourke T., Dale R., Humer E., Probst T., Pieh C. (2024). The association between problematic smartphone use and mental health in Austrian adolescents and young adults. Healthcare.

[34] Oh W.-O., Heo Y.-J. (2024). Exploring the link between smartphone overdependence, depression, and suicidal behaviors through the mediating effect of lifestyle risk behaviors among South Korean adolescents: A cross-sectional study using National Big Data. J. Pediatr. Health Care.

[35] Alqahtani R.A., AlSaadi Z.S., Al-Qahtani Z.A., Al-Garni A.M., Shati A.A., Malik A.A., Al Jabbar I.S., Mahmood S.E. (2024). Smartphone use and its association with body image distortion and weight loss behaviours among adolescents in Saudi Arabia. Technol. Health Care.

[36] Wu H., Lin Y., Yang L., Lai W., Li Y., Xu Y., Wang W., Yang L., Lu C., Yan B. (2024). Association between changes in adherence to the 24-hour movement guidelines with depression and anxiety symptoms among Chinese adolescents: A prospective population-based study. Child Adolesc. Psychiatry Ment. Health.

[37] Nagamitsu S., Kanie A., Sakashita K., Sakuta R., Okada A., Matsuura K., Ito M., Katayanagi A., Katayama T., Otani R. (2022). Adolescent health promotion interventions using well-care visits and a smartphone cognitive behavioral therapy app: Randomized controlled trial. JMIR Mhealth Uhealth.

[38] Raknes S., Al-Khayat A., Schuler B. (2024). Digitalized social and emotional learning and better wellbeing among displaced Syrian adolescents in Lebanon. Int. J. Ment. Health.

[39] Zhou X., Edirippulige S., Jones A., Bai X., Smith A.C., Bambling M. (2023). The feasibility, acceptability and efficacy of an app-based intervention (the Coping Camp) in reducing stress among Chinese school adolescents: A cluster randomised controlled trial. PLoS ONE.

[40] Feng S., Liu R., Jung Y., Barry A., Park J. (2024). Sex differences among U.S. high school students in the associations of screen time, cyberbullying, and suicidality: A mediation analysis of cyberbullying victimization using the youth risk behavioural surveillance survey 2021. J. Community Appl. Soc. Psychol..

[41] Hamilton J.L., Dalack M., Boyd S.I., Jorgensen S., Dreier M.J., Sarna J., Brent D.A. (2024). Positive and negative social media experiences and proximal risk for suicidal ideation in adolescents. J. Child Psychol. Psychiatry.

[42] Mougharbel F., Chaput J.-P., Sampasa-Kanyinga H., Hamilton H.A., Colman I., Leatherdale S.T., Goldfield G.S. (2023). Heavy social media use and psychological distress among adolescents: The moderating role of sex, age, and parental support. Front. Public Health.

[43] Cohen D.A., Zarr R., Estrada E., Zhong H., Han B. (2025). Association of children’s electronic media use with physical activity, cognitive function, and stress. Prev. Med..

[44] Engberg E., Hietajärvi L., Maksniemi E., Lahti J., Lonka K., Salmela-Aro K., Viljakainen H. (2022). The longitudinal associations between mental health indicators and digital media use and physical activity during adolescence: A latent class approach. Ment. Health Phys. Act..

[45] Han M. (2023). The prediction model of body image distortion in Korean adolescent in the era of covid-19 using decision tree analysis. Res. Community Public Health Nurs..

[46] Xiang M., Liu Y., Yamamoto S., Mizoue T., Kuwahara K. (2022). Association of changes of lifestyle behaviors before and during the COVID-19 pandemic with mental health: A longitudinal study in children and adolescents. Int. J. Behav. Nutr. Phys. Act..

[47] Freitas B.H., Gaíva M.A., Diogo P.M., Bortolini J. (2022). Relationship between lifestyle and self-reported smartphone addiction in adolescents in the COVID-19 pandemic: A mixed-methods study. J. Pediatr. Nurs..

[48] Tao Y., Tang Q., Zou X., Wang S., Ma Z., Zhang L., Liu X. (2023). Effects of attention to negative information on the bidirectional relationship between fear of missing out (FOMO), depression and smartphone addiction among secondary school students: Evidence from a two-wave moderation network analysis. Comput. Hum. Behav..

[49] Yu L., Du M. (2022). Social networking use, mental health, and quality of life of Hong Kong adolescents during the COVID-19 pandemic. Front. Public Health.

[50] Liu J., Liu R.-D., Ding Y., Hong W., Yang Y. (2024). Beyond the wish: Actual mobile phone use surpassing desire brings more distress. Int. J. Ment. Health Addict..

[51] Smout S., Champion K.E., O’Dean S., Halladay J., Gardner L.A., Newton N.C. (2024). Adolescent lifestyle behaviour modification and mental health: Longitudinal changes in diet, physical activity, sleep, screen time, smoking, and alcohol use and associations with psychological distress. Int. J. Ment. Health Addict..

[52] Agai M.S. (2022). Disconnectivity synced with identity cultivation: Adolescent narratives of digital disconnection. J. Comput.-Mediat. Commun..

[53] Ciudad-Fernández V., Zarco-Alpuente A., Escrivá-Martínez T., Herrero R., Baños R. (2024). How adolescents lose control over social networks: A process-based approach to problematic social network use. Addict. Behav..

[54] Kjellenberg K., Ekblom O., Ahlen J., Helgadóttir B., Nyberg G. (2022). Cross-sectional associations between physical activity pattern, sports participation, screen time and mental health in Swedish adolescents. BMJ Open.

[55] Yun H., Choi E.K. (2024). Association between smartphone overdependence and mental health in South Korean adolescents: A secondary data analysis. Child Health Nurs. Res..

[56] Anthony R., Young H., Hewitt G., Sloan L., Moore G., Murphy S., Cook S. (2022). Young people’s online communication and its association with mental well-being: Results from the 2019 student health and well-being survey. Child Adolesc. Ment. Health.

[57] Badesha K., Wilde S., Dawson D.L. (2022). Mental Health Mobile Application self-help for adolescents exhibiting psychological distress: A single case experimental design. Psychol. Psychother. Theory Res. Pract..

[58] Du X., Ding C., Xiang G., Duan H., Chen J., Chen H. (2024). Mediation of expressive suppression and emotional well-being in the relationship between loneliness and mobile phone addiction among Chinese adolescents. Child. Youth Serv. Rev..

[59] Yang C., Pham T., Ariati J., Smith C. (2023). Well-being implications of digital social multitasking in Adolescent friendship: A latent profile analysis. Cyberpsychol. Behav. Soc. Netw..

[60] Kim A.-R., Lee S., Park J.-H. (2022). An analysis of the factors affecting children and adolescent lifestyle in South Korea: A cross-sectional study with KCYPS 2018. PLoS ONE.

[61] Kwon S., Kim R., Lee J.-T., Kim J., Song S., Kim S., Oh H. (2022). Association of smartphone use with body image distortion and weight loss behaviors in Korean adolescents. JAMA Netw. Open.

[62] Livet A., Boers E., Laroque F., Afzali M.H., McVey G., Conrod P.J. (2022). Pathways from adolescent screen time to eating related symptoms: A multilevel longitudinal mediation analysis through self-esteem. Psychol. Health.

[63] Yu J.J., Meng X. (2024). Interplay of mobile phone dependency and catch-up sleep in South Korean youth: A seven-wave study of two nationally representative cohorts. Comput. Hum. Behav..

[64] Kim E., Lee K. (2022). Relationship between smartphone addiction and sleep satisfaction: A cross-sectional study on Korean adolescents. Healthcare.

[65] Kurudirek F., Baş N.G., Arıkan D. (2024). The relationship between sleep quality and smartphone addiction among adolescents. Sağlık Bilim. Üniversitesi Hemşirelik Derg..

[66] Vézina-Im L.A., Beaulieu D., Turcotte S., Roussel-Ouellet J., Labbé V., Bouchard D. (2022). Association between recreational screen time and sleep quality among adolescents during the third wave of the COVID-19 pandemic in Canada. Int. J. Environ. Res. Public Health.

[67] Chaveepojnkamjorn W., Srikaew J., Satitvipawee P., Pitikultang S., Khampeng S. (2023). Association between media use and poor sleep quality among senior high school students: A cross-sectional study. F1000Research.

[68] Pillion M., Gradisar M., Bartel K., Whittall H., Kahn M. (2022). What’s “app”-ning to adolescent sleep? Links between device, app use, and sleep outcomes. Sleep Med..

[69] Reichenberger D.A., Master L., Mathew G.M., Snyder C.K., Buxton O.M., Hale L., Chang A.-M. (2024). Interactive screen-based activities predict worse actigraphic sleep health that night among adolescents. J. Adolesc. Health.

[70] Lafontaine-Poissant F., Lang J.J., McKinnon B., Simard I., Roberts K.C., Wong S.L., Chaput J.P., Janssen I., Boniel-Nissim M., Gariépy G. (2024). Social media use and sleep health among adolescents in Canada. Health Promot. Chronic Dis. Prev. Can. Res. Policy Pract..

[71] Brosnan B., Haszard J.J., Meredith-Jones K.A., Wickham S.-R., Galland B.C., Taylor R.W. (2024). Screen use at bedtime and sleep duration and quality among youths. JAMA Pediatr..

[72] Gaya A.R., Brum R., Brites K., Gaya A., de Borba Schneiders L., Duarte Junior M.A., López-Gil J.F. (2023). Electronic device and social network use and sleep outcomes among adolescents: The EHDLA study. BMC Public Health.

[73] Nagata J.M., Cheng C.M., Shim J., Kiss O., Ganson K.T., Testa A., He J., Baker F.C. (2024). Bedtime screen use behaviors and sleep outcomes in early adolescents: A prospective cohort study. J. Adolesc. Health.

[74] Reardon A., Lushington K., Agostini A. (2023). Adolescent sleep, distress, and technology use: Weekday versus weekend. Child Adolesc. Ment. Health.

[75] Tkaczyk M., Lacko D., Elavsky S., Tancoš M., Smahel D. (2023). Are smartphones detrimental to adolescent sleep? An electronic diary study of evening smartphone use and sleep. Comput. Hum. Behav..

[76] Kiss O., Nagata J.M., de Zambotti M., Dick A.S., Marshall A.T., Sowell E.R., Van Rinsveld A., Guillaume M., Pelham W.E., Gonzalez M.R. (2023). Effects of the COVID-19 pandemic on screen time and sleep in early adolescents. Health Psychol..

[77] Daniels A., Pillion M., Rullo B., Mikulcic J., Whittall H., Bartel K., Kahn M., Gradisar M., Bauducco S.V. (2023). Technology use as a sleep-onset aid: Are adolescents using apps to distract themselves from negative thoughts?. Sleep Adv..

[78] Yue L., Cui N., Jiang L., Cui N. (2023). Screen use before sleep and emotional problems among adolescents: Preliminary evidence of mediating effect of chronotype and social jetlag. J. Affect. Disord..

[79] Bainton J., Hayes B. (2022). Sleep in an at risk adolescent group: A qualitative exploration of the perspectives, experiences and needs of youth who have been excluded from mainstream education. Inq. J. Med. Care Organ. Provis. Financ..

[80] Burnell K., George M.J., Jensen M., Hoyle R.H., Odgers C.L. (2022). Associations between adolescents’ daily digital technology use and sleep. J. Adolesc. Health.

[81] Li B., Gu Y., Yang Y., Zhao M., Dong Y. (2023). The effect of problematic smartphone use on school engagement and disengagement among middle school students: The mediating role of academic procrastination and sleep quality. J. Adolesc..

[82] Bani-Issa W., Radwan H., Saqan R., Hijazi H., Fakhry R., Alameddine M., Naja F., Ibrahim A., Lin N., Naing Y.T. (2023). Association between quality of sleep and screen time during the COVID-19 outbreak among adolescents in the United Arab Emirates. J. Sleep Res..

[83] Burnell K., Garrett S.L., Nelson B.W., Prinstein M.J., Telzer E.H. (2024). Daily links between objective smartphone use and sleep among adolescents. J. Adolesc..

[84] Tamura N., Okamura K. (2024). Longitudinal course and outcome of social jetlag in adolescents: A 1-year follow-up study of the adolescent sleep health epidemiological cohorts. J. Sleep Res..

[85] Cho Y., In H., Park M., Park E.C., Kim S.H. (2023). Association of smartphone use with abnormal social jetlag among adolescents in Korea before and after COVID-19. Addict. Behav..

[86] Malik F. (2022). Developmental Stages of Social Emotional Development in Children.

[87] Mancinelli E., Ruocco E., Napolitano S., Salcuni S. (2022). A network analysis on self-harming and problematic smartphone use—The role of self-control, internalizing and externalizing problems in a sample of self-harming adolescents. Compr. Psychiatry.

[88] Tuncay S., Sarman A. (2023). The relationship between smartphone and computer games and anger in adolescents. Marmara Med. J..

[89] Charlton J., Malik I., Ashley A.M., Newton A., Toombs E., Schmidt F., Olthuis J.V., Stasiuk K., Bobinski T., Mushquash A. (2025). Identifying the minimal clinically important difference in emotion regulation among youth using the JoyPop app: Survey study. JMIR Form. Res..

[90] Sina E., Buck C., Ahrens W., Coumans J.M.J., Eiben G., Formisano A., Lissner L., Mazur A., Michels N., Molnar D. (2023). Digital media exposure and cognitive functioning in European children and adolescents of the I. Family study. Sci. Rep..

[91] Armstrong-Carter E., Garrett S.L., Nick E.A., Prinstein M.J., Telzer E.H. (2023). Momentary links between adolescents’ social media use and social experiences and motivations: Individual differences by peer susceptibility. Dev. Psychol..

[92] Cheng J., Peng C., Rong F., Wang Y., Tan Y., Yu Y. (2024). Mobile phone addiction and suicide behaviors among Chinese adolescents: The mediation of poor sleep quality. J. Behav. Addict..

[93] Chory A., Callen G., Nyandiko W., Njoroge T., Ashimosi C., Aluoch J., Scanlon M., McAteer C., Apondi E., Vreeman R. (2022). A pilot study of a mobile intervention to support mental health and adherence among adolescents living with HIV in western Kenya. AIDS Behav..

[94] Tarabay R., Gerges S., Sarray El Dine A., Malaeb D., Obeid S., Hallit S., Soufia M. (2023). Exploring the indirect effect of loneliness in the association between problematic use of social networks and cognitive function in Lebanese adolescents. BMC Psychol..

[95] Roberts T.-A., Daniels E., Weaver J., Zanovitch L. (2022). “Intermission!” A short-term social media fast reduces self-objectification among pre-teen and teen dancers. Body Image.

[96] Brand C., Fochesatto C.F., Gaya A.R., Schuch F.B., López-Gil J.F. (2024). Scrolling through adolescence: Unveiling the relationship of the use of social networks and its addictive behavior with psychosocial health. Child Adolesc. Psychiatry Ment. Health.

[97] Mao N., Li T., Li C., Ding R., Zhang Q., Cui L. (2023). Smartphone-based training of cognitive bias modification: Efficacy for reducing social anxiety in Chinese adolescents. J. Child Fam. Stud..

[98] Poujol M.C., Pinar-Martí A., Persavento C., Delgado A., Lopez-Vicente M., Julvez J. (2022). Impact of mobile phone screen exposure on adolescents’ cognitive health. Int. J. Environ. Res. Public Health.

[99] da Silva N.S., Campos L.B., de Rocha M.M., Carreiro L.R. (2023). Electronic media and symptoms of inattention/hyperactivity among children/adolescents during the COVID-19 pandemic. Psicol.—Teor. E Prática.

[100] Stieger S., Wunderl S. (2022). Associations between social media use and cognitive abilities: Results from a large-scale study of adolescents. Comput. Hum. Behav..

[101] Wallace J., Boers E., Ouellet J., Afzali M.H., Conrod P. (2023). Screen time, impulsivity, neuropsychological functions and their relationship to growth in adolescent attention-deficit/hyperactivity disorder symptoms. Sci. Rep..

[102] Gao L., Zhao W., Caselli G., Zhang Y., Wang X., Zhang Y., Chen H. (2024). Weak and interfered self-control fails to block problematic mobile phone use: The role of craving and desire thinking. J. Psychiatr. Res..

[103] Sennock S., v. Lieres und Wilkau K., Günther A., Brandhorst I., Zinke K., Conzelmann A., Renner T.J., Kurz E.-M. (2024). Investigation of the influence of 45-min pre-sleep social media use on sleep quality and memory consolidation in adolescents. Sleep Med..

[104] Ng M.Y., Harrison A., Bath E., Kemp K., Galbraith K., Brown L.K., Tolou-Shams M. (2022). Sexting and behavioral health in first-time justice-involved adolescents. Child. Youth Serv. Rev..

[105] Fabio R.A., Suriano R. (2023). The influence of smartphone use on tweens’ capacity for complex critical thinking. Children.

[106] Fabio R.A., Suriano R. (2024). The role of smartphone use in sensory processing: Differences between adolescents with ADHD and typical development. Int. J. Environ. Res. Public Health.

[107] Palejwala A.H., O’Connor K.P., Milton C.K., Anderson C., Pelargos P., Briggs R.G., Conner A.K., O’Donoghue D.L., Glenn C.A., Sughrue M.E. (2020). Anatomy and white matter connections of the fusiform gyrus. Sci. Rep..

[108] Lewis R.G., Florio E., Punzo D., Borrelli E. (2021). The brain’s reward system in health and disease. Circadian Clock in Brain Health and Disease; Advances in Experimental Medicine and Biology.

[109] Montag C., Becker B. (2023). Neuroimaging the effects of smartphone (over-) use on brain function and structure—A review on the current state of MRI-based findings and a roadmap for future research. Psychoradiology.

[110] Wacks Y., Weinstein A.M. (2021). Excessive Smartphone Use Is Associated with Health Problems in Adolescents and Young Adults. Front. Psychiatry.

